# Hydrodynamic metasurface for programming electromagnetic beam scanning on the Azimuth and elevation planes

**DOI:** 10.1038/s41378-022-00371-5

**Published:** 2022-04-21

**Authors:** Aqeel Hussain Naqvi, Sungjoon Lim

**Affiliations:** School of Electrical and Electronics Engineering, Chung-Ang University, Seoul, 06974 Republic of Korea

**Keywords:** Nanophotonics and plasmonics, Electrical and electronic engineering

## Abstract

The development of multifunctional and reconfigurable metasurfaces capable of manipulating electromagnetic waves has created new opportunities for various exciting applications. Extensive efforts have been applied to exploiting active metasurfaces with properties that can be controlled by externally controlling active components. However, previous approaches have poor switch isolation, power handling limitations due to nonlinear effects, and complex biasing networks. Therefore, dynamically tunable metasurfaces have become a burgeoning field in many research areas. This paper reports a hydrodynamic metasurface (HMS) that can be programmed to realize electromagnetic beam scanning on the azimuth and elevation planes. The proposed HMS platform incorporates four micropumps, each controlling four metasurface elements via microfluidic channels, built into the HMS base. The proposed platform regulates microfluidic flow through micropumps, causing irregularities in incident wave transmission phase. An HMS was built as a proof of concept, and far-field scanning experiments were performed. Numerical and experimental results verify the feasibility of electromagnetic beam scanning using a hydrodynamic metasurface. This work advances metasurface research, with very high potential for wide-ranging application and a promising route for replacing bulky cascading active components.

## Introduction

Metamaterials have established a role as interesting research subjects because they exhibit exotic electromagnetic (EM) behaviors, attracting widespread attention from physics and engineering disciplines. Metasurfaces are the two-dimensional (2D) counterparts of metamaterials and have been intensively investigated because of their powerful ability to control EM waves due to compactness, low-cost, high-level integration, and ease of fabrication. Many metasurface designs have been reported for diverse applications including reconfigurable radiating structures^1–4^, polarization converters^5–7^, EM cloaks^8–10^, vortex beam generators^11–13^, hologram generators^14–17^, and beam deflectors^18–22^.

Rapid integrated communication system developments, particularly in radar applications, where precise target information is crucial, have set new standards for scanning systems, requiring full scanning coverage in both the azimuth and elevation planes while keeping costs low and preserving design simplicity. Hence, considerable effort has been applied to achieving more accurate angle information. Yuk et al.^23^ proposed a mechanically rotatable metasurface with a continuous radiating beam. Beam steering through 0°–12° was achieved by modifying the liquid crystal molecules from nematic to isotropic through voltage biasing^24^. Hashemi et al.^25^ achieved a beam steering angle of 44° using VO2-based metasurfaces in the horizontal and vertical directions.

Multiple EM functionalities can be achieved with reconfigurable and programmable (active) metasurfaces^20–33^. Active component interactions with metamaterials introduce transmission phase inconsistencies because of partial reflection off the metasurface^34–36^, enabling reconfigurable beam steering^37–39^. Wen et al.^40^, introduced additional degrees of freedom for advanced EM wave manipulation in space-time and frequency domains by spatially integrating reconfigurable microelectromechanical systems (MEMS) and photoresponsive material into metamaterials. Active tuning using PIN diodes, varactor diodes, barium strontium titanate (BST) devices, and MEMS switching has been incorporated in many reconfigurable metasurface designs. However, cascading active components and phase shifters not only make the entire system cumbersome, preventing it from being miniaturized, but also make it difficult to integrate. Digitally coded programmable metasurfaces were proposed in 2014 to digitally manipulate and control EM waves^41^. In contrast, with conventional metasurfaces characterized by continuous parameters, coded metasurfaces are controlled digitally in a programmable fashion, allowing more complex designs and functional variety to be implemented. Zhang et al.^11^ proposed a multifunctional coding metasurface with integrated transmission and reflection functions for full spatial control of EM waves.

Compared to semiconductor and MEMS switching^42^, fluidic tuning has attracted extensive attention due to its unique advantages, such as being mechanically based and highly linear, making it the optimum choice for high-power microwave applications. Second, fluidic devices make use of soft materials and standard technologies to create tunable devices that are flexible and wearable. Microfluidics have been integrated into various applications, including vibration^43^, tilt^44^, and tactile sensors^45,46^. Similarly, metamaterials with dynamically modifiable properties would be ideal for many metasurface/metamaterial structures. Various materials, including eutectic gallium indium (EGaIn)^47^, mercury^48^, liquid crystal^24,49,50^, and water^51^, have been utilized to realize frequency^52^, polarization^53^, and radiation pattern^54^ reconfigurability. Perhaps surprisingly, water is often a good candidate for fluidic solutions, as it is the most abundant fluid on earth, easily available, environmentally friendly, and low cost.

**Fig. 1:**
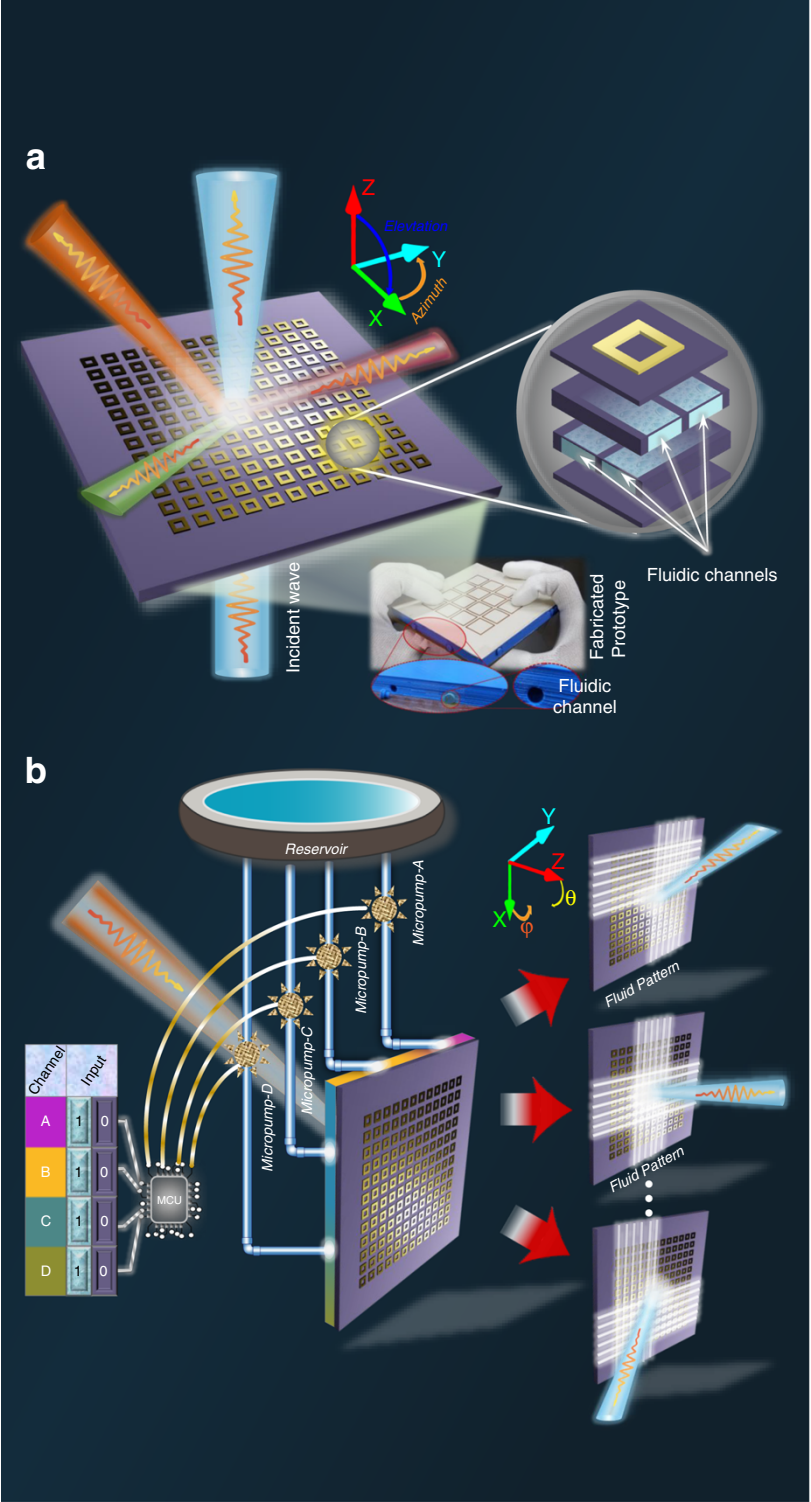
Proposed hydrodynamic metasurface (HMS) concept and design. **a**, **b** Example demonstrating full-space EM-wave control using programmable fluid flow inside fluidic channels. Beam scanning is realized by gradient phase transformation of incident waves after they pass through the HMS. The proposed HMS platform incorporates four micropumps, each controlling four metasurface elements via four microfluidic channels built into the HMS base.

Our proposed work implies a new “hydrodynamic coding concept” using a hydrodynamic metasurface (HMS). Nevertheless, realistic demonstrations of metasurfaces basically require fine discretization of the structures to achieve a changeable phase response. Figure 1 shows the proposed programmable EM beam scanning in both the azimuth and elevation planes using an HMS. Here, phase transformation is achieved through binary programmable high dielectric fluid (water) flow using microcontroller-based microfluidic pumping inside microfluidic channels built into the HMS base. It is well-known that DI water has lower dielectric loss than tap water. However, in this work, it is observed that the radiation efficiency is not significantly decreased in DI water. The proposed concept is low cost, biofriendly, and ecologically compatible compared with active metasurfaces, and to the best of our knowledge, no similar concept has been previously reported for controlling EM waves in full space.

## Results

### Concept and design

Active component-based metasurfaces have poor switch isolation, power handling limitations due to nonlinear effects, and complex biasing networks. Microfluidics have been integrated into a variety of applications such as vibration sensing^43^, tilt sensing^44^, and tactile sensing^45^ due to their unique advantages compared with other tuning techniques. Likewise, if the properties of a metamaterial can be changed dynamically, it could possibly become the gold standard for metasurface/metamaterial structures. Because of their various fascinating properties, such as being fluid at room temperature, various materials such as EGaIn, mercury, and liquid crystal polymers have been utilized to reconfigure RF properties. Water, an abundant, promptly available, and environmentally friendly resource, is typically not included among these fluids. However, due to the benefits of being low cost, readily available, and ecologically compatible, water has become the most well-known. Because of the high sensitivity of conductive or electrolytic fluids, they can be used for tilt or vibration sensors. On the other hands, dielectric fluids are less sensitive to vibrations/tilting, as they maintain an isolation barrier between the electrodes.

Considering these advantages, we introduce a new “hydrodynamic coding concept” using a HMS. The proposed concept provides three-dimensional (3D) beam scanning capabilities through the programmable injection of water using micropumps and a controller board. The proposed metasurface does not require a complex biasing network and can be programmed to realize EM beam scanning on the azimuth and elevation planes. This work advances metasurface research, with very high potential for wide-ranging application and a promising route for replacing bulky cascading active components.

### Hydrodynamic metacell analysis

**Fig. 2:**
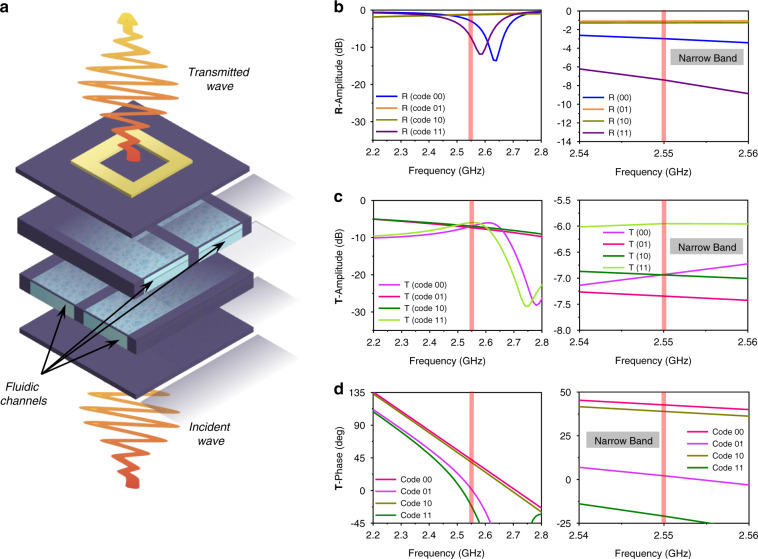
Analysis of the hydrodynamic metacell with periodic boundaries. **a** Exploded view of HMS metacell, including the top conductive square ring element printed on the dielectric substrate and 3D printed fluidic channels in the *xz* and *yz* planes in the HMS base. Amplitudes of (**b**) the reflection coefficient (R), (**c**) transmission coefficient (T), and (**d**) transmission phase for the metacell in the four states.

The proposed HMS is a 2D metacell lattice based on the phase changing surface concept. Figure 2(a) shows the proposed reconfigurable HMS metacell 3D geometry, which can hydrodynamically change or manipulate the transmitted EM wave phase. We consider the metasurface of a programmable HMS with an array of conductive elements printed on top, two fluidic channels along the *x*-direction (channels A and B), and two fluidic channels along the *y*-direction (channels C and D) in the HMS base. A larger number of microfluidic channels can achieve a higher beam scanning resolution. Nevertheless, we use four microfluidic channels because, in this work, we use an MP6-QuadEVA microcontroller, which facilitates the simultaneous control of up to four MP6 micropumps.

Therefore, we use four microfluidic channels so that the microfluidic channels can be controlled with a single microcontroller. The fluidic channel state can be either empty or water-filled, i.e., binary, and we set 0 = empty and 1 = water-filled channel. In contrast to traditional active metasurfaces that use active components and complex biasing networks to control EM fields, we propose a programmable HMS that simply manipulates EM waves using the channel coding to represent different fluidic channel states. The channel states are toggled from 0 to 1 (or vice versa) to steer transmitted waves from normally incident EM waves in the elevation and azimuth planes. Thus, the proposed HMS can control the EM phase response and realize versatile 3D wave manipulation by programming various channel codes.

Fluidic channels were created using 3D printing on a polylactic acid (PLA) substrate with dielectric constant = 2.5 and dielectric loss tangent = 0.02. EM wave phase variation can be realized hydrodynamically using water flow inside the fluidic channels controlled by programable electronic pumping. The dielectric permittivity and dielectric loss tangent for water (77.66 and 0.16, respectively) were derived using the probe-kit setup Agilent Keysight N1501A and used for full-wave simulation of the proposed geometry. Simulations applying the Floquet port theorem were used to analyze the hydrodynamic metacell transmission and reflection characteristics, where the metacells were characterized in four states: 00, 01, 10, and 11.

Figure 2(b, c) presents the reflection and transmission coefficient magnitudes for the four states. The reflection coefficient magnitudes at 2.55 GHz are −2.9, −1.3, −1.4, and −7.4 dB and the transmission coefficient magnitudes at 2.55 GHz are −6.9, −7.3, −6.8, and −6.1 dB for channel codes 00, 01, 10, and 11, respectively. Figure 2(d) shows the transmission phase characteristics of the proposed metacell in the four states. The transmission phases at 2.55 GHz are 42.8°, 1.7°, 38.2°, and 21° at 2.55 GHz for channel codes 00, 01, 10, and 11, respectively. In addition, their frequency responses in the narrow band are shown on the right side of each plot. As we can see, code 00 and code 11 show different amplitude trends because of different permittivities and dielectric loss tangents. It is observed that code 01 and code 10 exhibit similar reflection amplitude trends. However, their transmission amplitude and phase are different, as shown in Fig. 2(c, d). In addition, it is observed that code 00 and code 10 have similar amplitude trends for reflection and transmission coefficients. However, the transmission phase difference between code 00 and code 10 at 2.55 GHz is 4.6°, as shown in Fig. 2(d). Note that the variation in effective permittivity generates more phase variation than amplitude variation. In addition, the phase variation is more important for beam scanning under partial reflection and partial transmission.

### Hydrodynamic metasurface analysis

Consider the metasurface as a 2D planar array of radiating elements stimulated by a signal of distinct phase and magnitude. Elements are identically ordered along a 2D grid in the *xy* plane with element gap *p* in the *x*- and *y*-directions. Therefore, the far-field pattern for the 2D planar array can be expressed as [Eq. 1]

1 $$F\left( {\theta ,\varphi } \right) = \mathop {\sum}\limits_{m = 1}^N {e\,^{j\frac{{2\pi }}{\lambda }\left( {m{{{\mathrm{sin}}}}\theta {{{\mathrm{cos}}}}\varphi + \beta _x} \right)}} \mathop {\sum}\limits_{n = 1}^N {e^{j\frac{{2\pi }}{\lambda }\left( {nd_y{{{\mathrm{sin}}}}\theta {{{\mathrm{sin}}}}\varphi + \beta _y} \right)}}$$

where *N* is the number of array elements in the *x*- and *y*-directions, respectively; *p* is the array element periodicity; and β_*x*_ and β_*y*_ are progressive array element phase shifts in the *x*- ^and *y*^-directions, respectively. The normalized array factor form for (1) can be expressed as

2 $$AF\left( {\theta ,\varphi } \right) = \frac{1}{N}\left\{ {\frac{{{{{\mathrm{sin}}}}\left( {N\frac{{{{\Psi }}_x}}{2}} \right)}}{{{{{\mathrm{sin}}}}\left( {\frac{{{{\Psi }}_x}}{2}} \right)}}} \right\}\left\{ {\frac{{{{{\mathrm{sin}}}}\left( {N\frac{{{{\Psi }}_y}}{2}} \right)}}{{{{{\mathrm{sin}}}}\left( {\frac{{{{\Psi }}_y}}{2}} \right)}}} \right\}$$

where:

3 $${{\Psi }}_{\chi} = k_op{{{\mathrm{sin}}}}\theta _o{{{\mathrm{cos}}}}\varphi _{o} + \beta _\chi$$

and

4 $${{\Psi }}_{y} = k_op{{{\mathrm{sin}}}}\theta _o{{{\mathrm{sin}}}}\varphi _{o} + \beta _y$$

Simulations were used to obtain the progressive phase shift *β* due to fluid movement inside the metacell using Floquet’s theorem, a function of the simulated S-parameter set (*S11*(*x,y*)*, S12*(*x,y*)*, S21*(*x,y*), and *S22*(*x,y*)). The phase constant is a function of *x* and *y* since the S-parameters are also direction dependent:

5 $$\beta = \frac{1}{p}\left| {{{{\mathrm{Im}}}}} \right.\left( {{{{\mathrm{cos}}}}^{ - 1}\left( {\frac{{1 - S_{11}S_{22} + S_{12}S_{21}}}{{2S_{21}}}} \right)} \right)$$

Since *F*(*θ,φ*) in (1) is the product of two linear array factors in the *x*- and *y*-directions, the main beam direction for the 2D planar array can be determined from linear array factors in the corresponding directions.

**Fig. 3:**
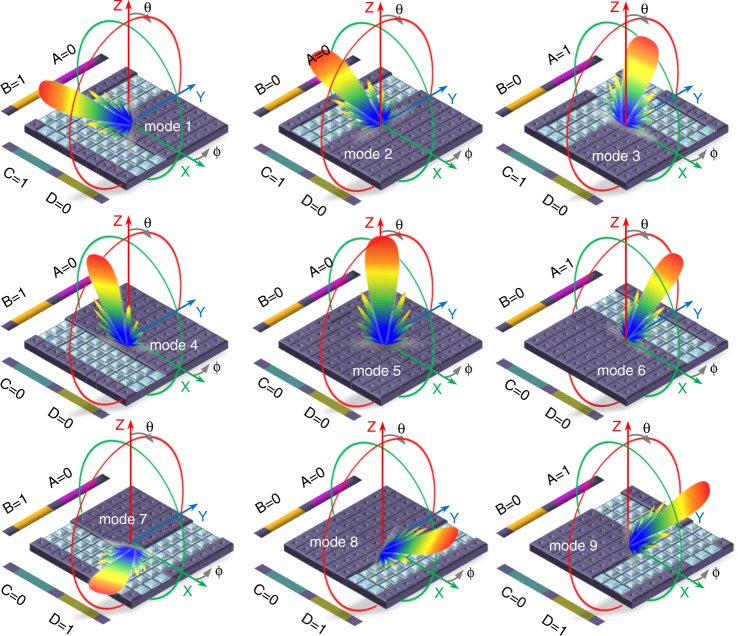
Binary coded HMS illustration showing the beam scanning capabilities of the coding sequences for modes 1–9, where A, B, C, and D represents the four microfluidic channels.

The 3D view and the HMS dimensions are shown in supplementary Fig. 1. Figure 3 presents binary-coded HMS illustrations to show beam scanning capabilities from the channel coding sequences for modes 1–9. We calculated the analytical beam pattern for mode 5 (coding sequence ABCD = 0000) when all channels are empty and normal transmission, i.e., steered to the broadside, from (2). Progressive phase shifts β_*x*_ and β_y_ are 0.184 and 0.318, respectively, according to metacell analysis using Floquet’s theorem with periodicity *p* = *λ/6*.

Supplementary Fig. 2 shows the analytical beam patterns for modes 7 and 9 calculated from (2) in the 2D normalized elevation and azimuth planes compared with the numerical radiation patterns for full-wave simulations generated by ANSYS HFSS at an operational frequency of 2.55 GHz for modes 5, 7, and 9. The main beam from the AF pattern is steered from the broadside to offset angles of ±22.5° in the elevation plane and ±57° in the azimuth plane, whereas they were expected at offset angles of ±20° and ±65° in the elevation and azimuth planes, respectively, from the numerical patterns.

### MP6-micropumping analysis

Previously, we injected fluid into microfluidic channels (100 × 0.51 mm, length × width)^55^, and 1–1.5 s was required to completely fill the microfluidic channel; hence, the infill rate = 66.7–100 mm/sec. In the current study, the microfluidic channel = 120 × 4 mm (length × diameter); hence, the estimated realistic switching time to completely fill channel A was 70–75 s.

We estimated the power budget for the proposed design as follows. Each micropump for water injection/extraction consumes 0.15 W (DC) and can switch four metacells, and the control board consumes 2.1 W during switching. The proposed design comprises 4 × 4 metacells, consuming 2.7 W total per switching state, significantly less than other electronic switching options. Fluidic technology is inappropriate for applications that require high switching speeds since larger metasurfaces require longer fluidic channels and hence longer injection time.

However, given ongoing fluidic technology developments, the proposed fluidic approach is a potential candidate for periodic structures that require many tunable components. Furthermore, water dielectric loss increases with increasing frequency; hence, there is much higher path loss at high frequencies. The loss in fluidic switching is determined by the fluid dielectric losses. The metasurface superstrate is a critical radiation region, and employing lossy material (i.e., water) increases device insertion loss, reducing radiation efficiency.

### Experimental verification

#### S-parameter measurement

The S-parameters for the fabricated prototype were measured using an Anritsu MS2038C network analyzer (Anritsu Corporation, Kanagawa Prefecture, Japan), with an MP6 micropump and MP6-QuadEVA microcontroller to switch between different modes via water injection/extraction from fluidic channels A, B, C, and D. Table 1 shows the nine discrete modes available for the proposed HMS depending on the different fluidic channel codes.

**Table 1:** Operational modes for the proposed HMS device and comparison between the numerical and experimental performance.

Mode	Channel value				Maximum beam scanning value			
	A	B	C	D	Elevation		Azimuth	
					Simulation	Measurement	Simulation	Measurement
1	0	1	1	0	26°	15°	252°	230°
2	0	0	1	0	−10°	0°	180°	190°
3	1	0	1	0	10°	20°	100°	100°
4	0	1	0	0	32°	30°	281°	270°
5	0	0	0	0	2°	10°	10°	0°
6	1	0	0	0	30°	20°	90°	90°
7	0	1	0	1	28°	30°	300°	300°
8	0	0	0	1	28°	10°	0°	0°
9	1	0	0	1	30°	20°	70°	50°

Supplementary Fig. 4 shows simulated and measured S-parameters for the proposed HMS in different modes.

#### Far-field pattern measurement

**Fig. 4:**
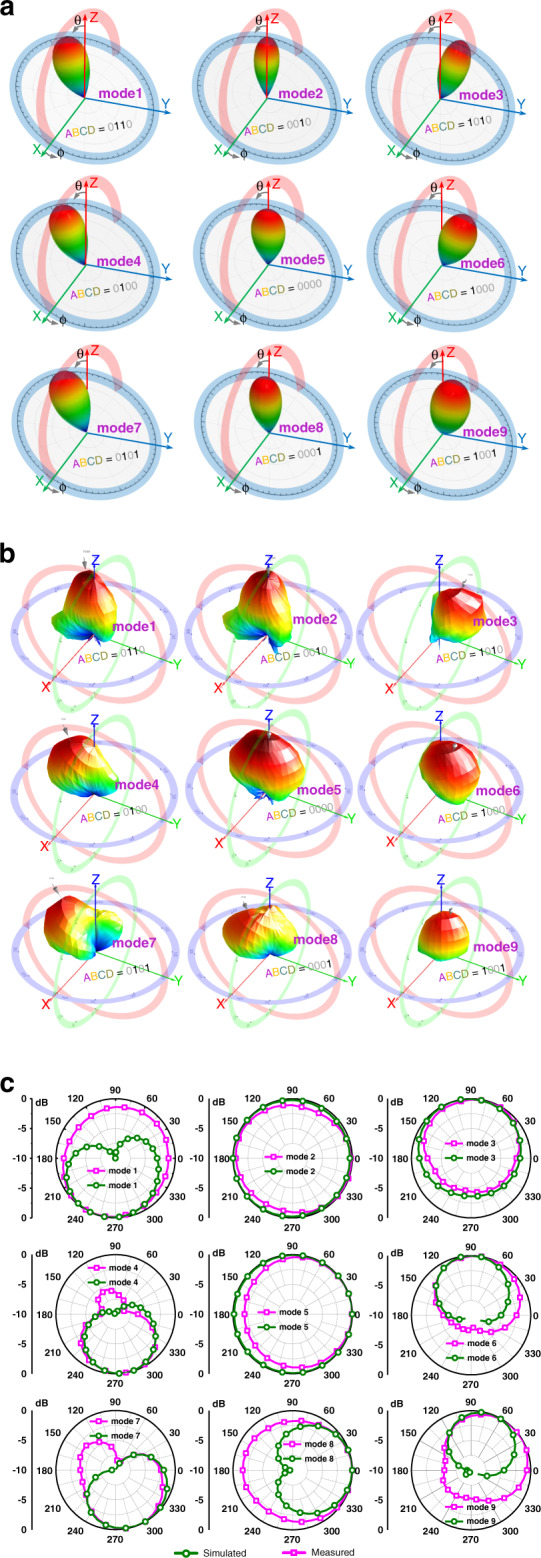
Simulated and measured two (2D) and three-dimensional (3D) far-field radiation patterns for the nine operational modes of the proposed hydrodynamic metasurface. **a** 3D simulated, (**b**) 3D measured, and (**c**) 2D simulated & measured.

The far-field radiation pattern for the fabricated metasurface prototype was measured using a commercial far-field measurement system in a shielded RF anechoic chamber. Figure 4 compares simulated and experimental results for 3D beam scanning. The experimental results were obtained by rotating the mounted HMS prototype to determine 3D radiation patterns at 2.55 GHz for all nine modes. The beam directions for modes 1, 4, and 7 were steered to 15°, 30°, and 30° from the broadside in the φ = 230°, 270°, and 300° planes, respectively, whereas the mode 3, 6, and 9 beam directions were all steered to 20° from the broadside in the φ = 100°, 90°, and 50° planes, respectively. Broadside radiation was obtained when all channels were toggled to code 0 (i.e., mode 5), and modes 2 and 8 beam directions were steered to 10° from the broadside in φ = 190° and 0° planes, respectively. The proposed metasurface realized nine distinct beam-steering states, individually selectable by programmable fluid flow inside the HMS fluidic channels.

## Discussion

This paper successfully demonstrated a programmable HMS to realize EM beam scanning on both azimuth and elevation planes using changeable transmission phase via water injection in fluidic channels for different binary-coded sequences. The facile HMS fabrication process produced a broadside EM beam that can be programmed in distinct azimuthal directions with ±25° elevation by controlling programmable water injection inside the channels. The simulated and measured results exhibit good agreement, with small differences due to fabrication and measurement error. For instance, because we manually assembled the Rogers substrate and 3D printed layer, misalignment was unavoidable. Moreover, to observe the effect of the repeatability of EM-wave manipulation by the flow of microfluidics, we tested the device repeatedly in mode 6, as shown in supplementary Fig 5(a–c). The experimental results exhibit good agreement with slight variation caused by minor fluid leakage during 3D rotation of the prototype. Since we used 3D printed knobs to seal the outlet of channels, the unproper sealing and 3D rotation of the prototype can cause fluid leakage during measurements. We simulated the HMS for mode 6 with channels half-filled and fully filled with dielectric fluid to demonstrate the variation in the simulation and experimental results due to measurement tolerance. It is observed that fluidic leakage can cause error in the scanning angle, although it is not critical in the reflection coefficient. These tolerances will be reduced for subsequent fabrications by employing high resolution 3D printing, efficient fluidic pumping, and careful assembly. The fabrication technique employed here has several advantages compared with conventional techniques for tunable or reconfigurable metasurfaces, which require large numbers of diodes and biasing circuits for tuning.

The switching time of the fluidic components can be improved by the actuator and fluidic material. For instance, a piezoelectric actuated micropump (mp5, Bartels Mikrotechnik GmbH) can be utilized to control the fluid flow within microfluidic channels^48^. The switching time can be improved by adjusting the flow rate through the applied voltage. In addition, the speed depends significantly on the viscosity of the pumped liquid; therefore, using a liquid with lower viscosity as the lubricating material will significantly enhance the speed^56^.

The proposed HMS provides an efficient mechanism to program EM waves in the microwave region using water with a low profile and cost-effective design. The precision/accuracy of beam steering are primarily determined by the precision of the fabrication process and the effective use of microfluidics for tuning. Slight fabrication tolerances, inadequate sealing, and a fluidic injection/extraction process may lead to the modification of the effective permittivity of the HMS, resulting in a loss of beam-steering precision. Phased array solutions have certain drawbacks including limited scanning coverage, complex planar configuration, large size, and obviously high fabrication cost^57^. Metasurfaces, which are the 2-D counterparts of metamaterials, have been intensively investigated due to their powerful ability to control EM waves owing to their compactness, high-level integration, and ease of fabrication^33,58^. However, the electronically tunable metamaterial antennas are expensive to fabricate due to the high-cost factor of PIN diodes. Compared to other reported works in the literature, the advantages of the proposed work include its simplified structure, avoidance of extensive switches or lumped elements, low cost, lack of biasing circuitry, and lack of performance disruption caused by biasing circuitry. Surveillance or radar applications can tolerate their slower switching time, and the proposed technique provides a promising route for replacing bulky cascading active components for future devices as fluidic technology matures.

## Methods

### Hydrodynamic metasurface fabrication

We fabricated a prototype HMS to experimentally verify the proposed concept. The HMS upper layer (0.508 mm thick) was produced by photolithography on an RO3003 substrate (ϵr = 3, tan δ = 0.0013) from the Rogers Corporation. The base layer of the HMS was 3D printed using PLA filament (ϵr = 2.5, tan δ = 0.02). We characterized the effective dielectric constant of the substrate from S-parameters. The effective dielectric constant of the substrate when all tubes are filled with water is equivalent to 3.9. The effective dielectric constant of the substrate when all tubes are empty is equivalent to 1.6. A 3D printer (Ultimaker-2+, Geldermalsen, Netherlands) was used for the fabrication of the HMS bottom layer from a PLA filament. Under PC control, sequential layers of PLA material were printed to design the fluidic channels inside the HMS bottom layer. The printing speed was set within the 40–100 mm/s range. The printer nozzle diameter was set to 0.4 mm, which enabled the printer to print successive layers with 20 µm thickness (Z-resolution) and 400 µm line width (X/Y-resolution). We designed the HMS with dimensions of 120 × 120 mm, which conforms to 1.02*λo* × 1.02*λo* at 2.55 GHz. The fabricated prototype of the proposed HMS is shown in the supplementary note in supplementary Fig 3.

### Simulation and experimentation

For the simulations, the ANSYS High Frequency Structure Simulator (HFSS) was used to optimize the design and perform full-wave simulation for far-field propagation analysis. The effective permittivity and dielectric loss tangent of water (77.66 and 0.16, respectively) were extracted using the probe-kit setup Agilent Keysight N1501A and used in the full-wave simulation of the proposed geometry. The S-parameters were measured using an Anritsu MS2038C network analyzer (Anritsu Corporation, Kanagawa Prefecture, Japan), whereas the far-field radiation pattern was measured with a commercial far-field measurement system in an RF anechoic chamber. Mode switching was accomplished using MP6 micropumps and an MP6-QuadEVA microcontroller (Bartels Mikrotechnik GmbH, Dortmund, Germany) to control up to four MP6 micropumps simultaneously to inject/extract water from fluidic channels A, B, C, and D, as shown in the supplementary note in supplementary Fig 3.

## Supplementary information

Supplementary Information
