# Supplementary material for: Hydrodynamic metasurface for programming electromagnetic beam scanning on the Azimuth and elevation planes
Source: Microsyst Nanoeng. 2022 Apr 21;8:43. doi: 10.1038/s41378-022-00371-5 (PMC9023569; doi:10.1038/s41378-022-00371-5)
Supplement: Supplementary file 1 — Supplementary Information [file 41378_2022_371_MOESM1_ESM.pdf]

Supplementary Information for

Hydrodynamic Metasurface for Programming Electromagnetic Beam Scanning on Azimuth and Elevation Planes

*by Aqeel Hussain Naqvi, Sungjoon Lim**

Supplementary Note 1: Configuration of the proposed HMS

In our proposed work, we designed a rectangular ring as a meta-cell element. Initially, the length of the rectangular-ring element is decided from the given resonant frequency by calculating the following equation:

$$L = \frac{c}{2f_r}\sqrt{\frac{2}{\varepsilon_r + 1}} \quad (1)$$

, where *c* denotes the speed of light, f_r is the resonant frequency, and ε_r is the permittivity of the material. Because the dimensions of the HMS meta-cell must be much smaller than free space wavelength (λ_o), we used the meta-cell's periodicity $p = a = b = 20$ mm (i.e., $p = \lambda_o/6$). The size of the channel (*r*) is decided by considering both 3D printing resolution and parametric study in full-wave simulation. A larger channel radius (*r*) results in lower effective permittivity of the cell because of higher portion of air in the empty channel. Therefore, the resonant frequency can be increased with a larger r. On the other hands, a larger channel radius (*r*) results in higher effective permittivity of the cell because of higher portion of water in the water-filled channel. Therefore, the resonant frequency can be decreased with a larger r, as shown in supplementary Figure 1(a). Moreover, a larger channel radius (r) is preferred for larger variations, as shown in supplementary Figure 1(b)-(e). In this work, we decided $r = 2$ mm which is the largest channel radius after considering both maximum beam scanning capability and 3D printable dimensions. Finally, we designed the HMS (120 mm × 120 mm, which conforms to $1.02\lambda_o \times 1.02\lambda_o$ at 2.55 GHz). The 3D view and the HMS dimensions are shown below in supplementary Figure 1(f) as the supplementary note.

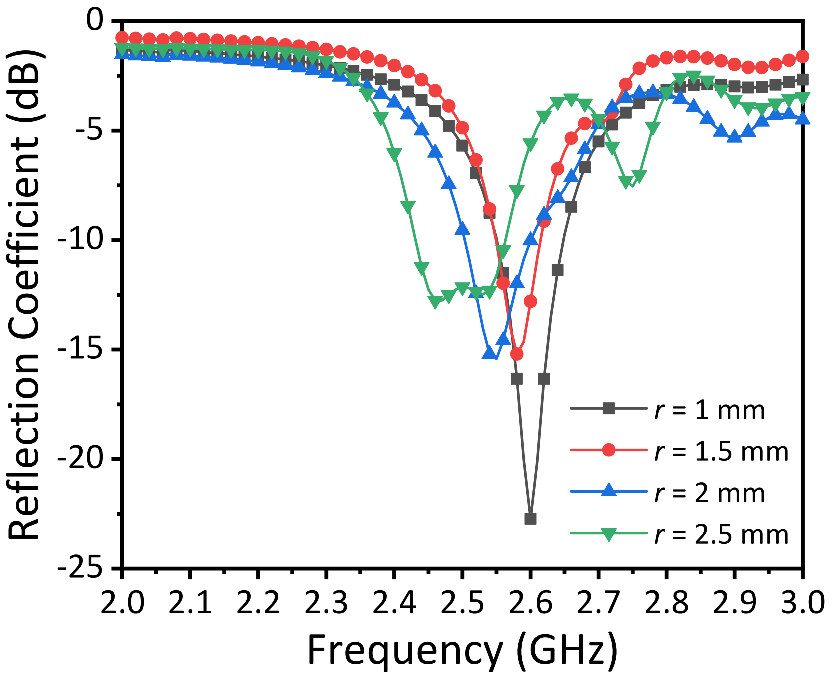

Reflection Coefficient (dB)
0
-5
-10
-15
-20
-25
2.0 2.1 2.2 2.3 2.4 2.5 2.6 2.7 2.8 2.9 3.0
Frequency (GHz)
r = 1 mm
r = 1.5 mm
r = 2 mm
r = 2.5 mm

(a)

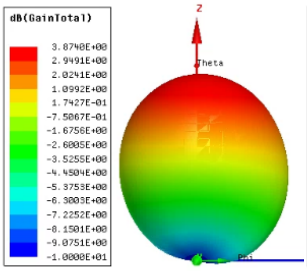

dB(GainTotal)

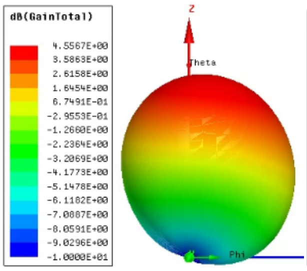

dB(GainTotal)

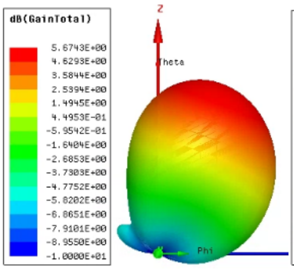

dB(GainTotal)

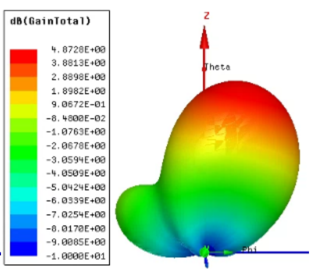

dB(GainTotal)

(b) (c) (d) (e)

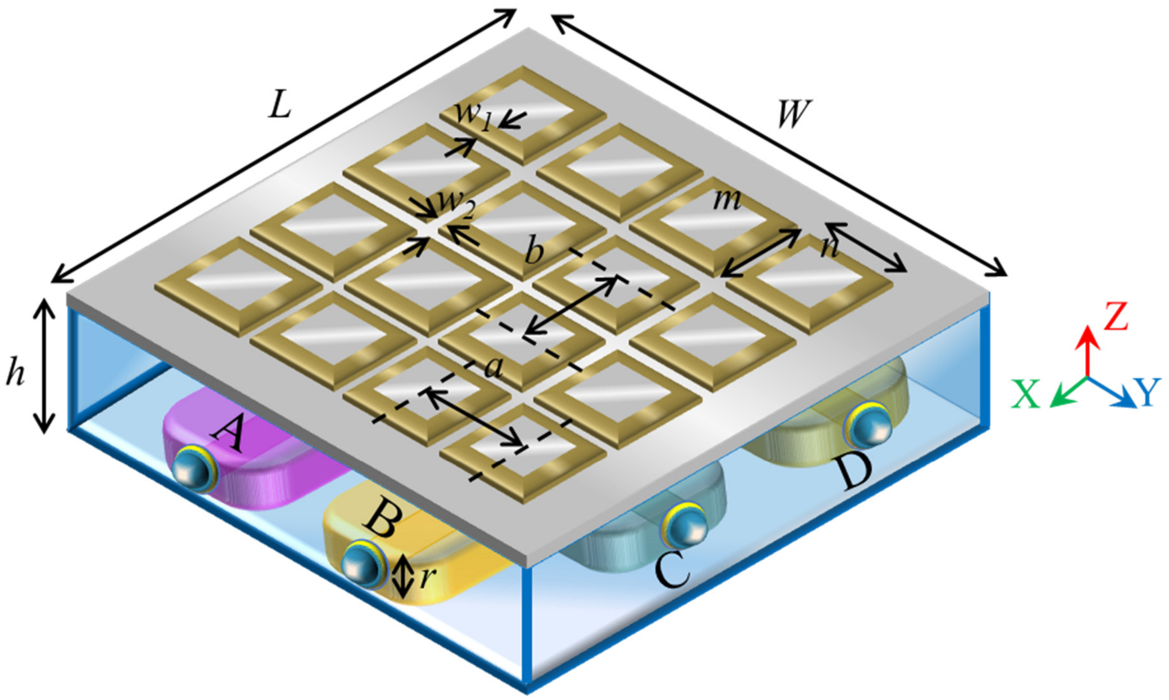

(f)

Supplementary Figure 1. Parametric analysis of the HMS in mode 6; (a) reflection coefficient plot with different values of r, and 3D radiation patterns at 2.55 GHz with; (b) r = 1 mm, (c) r = 1.5 mm, (d) r = 2 mm, and (e) r = 2.5 mm. (f) 3D view of proposed HMS prototype for the experimental proof-of-concept with dimensions: L=W=120, a=b=20, m=n=18, w_1=1, w_2=2, r=2, h=10.608. (Units: mm).

Supplementary Note 2: Comparison of Analytical and Numerical two-dimensional Beam-patterns

Supplementary Figure 1 shows the analytical beam patterns for modes 7 and 9 calculated from (2) in 2D normalized elevation and azimuth planes compared with numerical radiation patterns for full-wave simulations generated by ANSYS HFSS at operational frequency = 2.55 GHz for modes 5, 7, and 9. The main beam from the AF pattern is steered from the broadside to offset angle = ± 22.5° in the elevation and ± 57° in the azimuth plane, whereas they were expected at offset angles = ± 20° and ± 65° in the elevation and azimuth planes, respectively, from the numerical patterns.

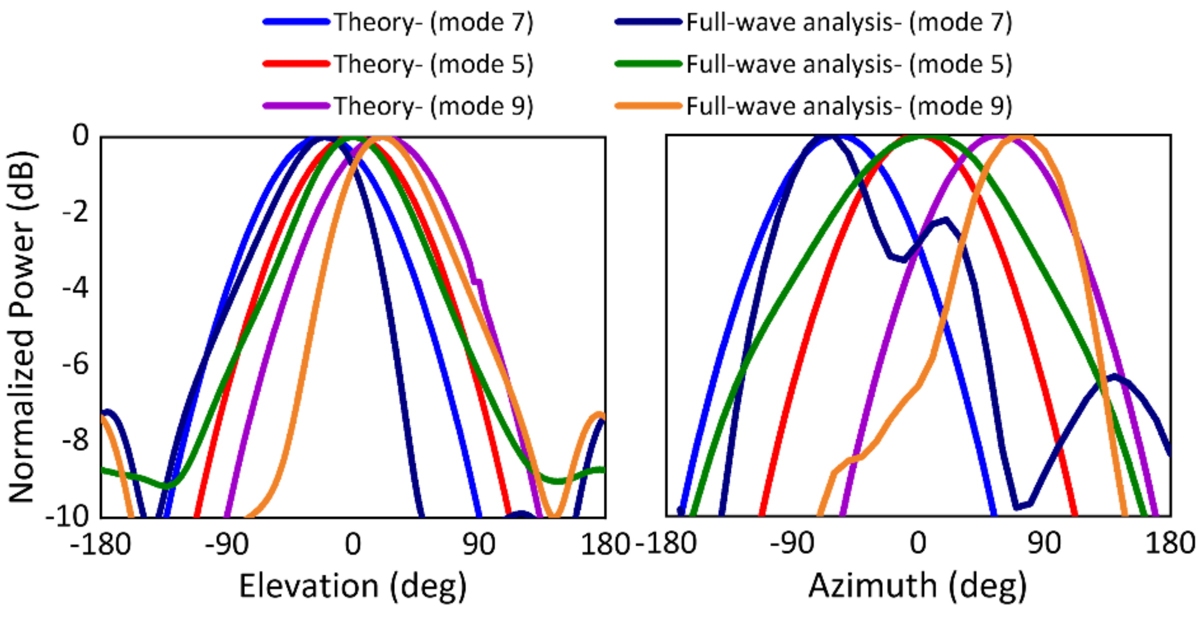

Supplementary Figure 2. Two-dimensional analytical results from (2) for HMS coding and full-wave simulation results from ANSYS HFSS for different coding sequences in elevation and azimuth planes.

Supplementary Note 3: Fabricated prototype of proposed hydrodynamic metasurface with MP6-microcontroller and micropumps

To experimentally demonstrate the presented idea a prototype of the HMS was fabricated. An Ultimaker-2+ 3D printer (Ultimaker-2+, Geldermalsen, Netherlands) was used for the fabrication of HMS bottom layer from a PLA filament. For the experimental demonstration, the injection/extraction procedure is carried out with the help of MP6 micropump and MP6-QuadEVA microcontroller (Bartels Mikrotechnik GmbH, Dortmund, Germany) that allows controlling of up to four MP6 micropumps simultaneously, as shown in Supplementary Figure 3.

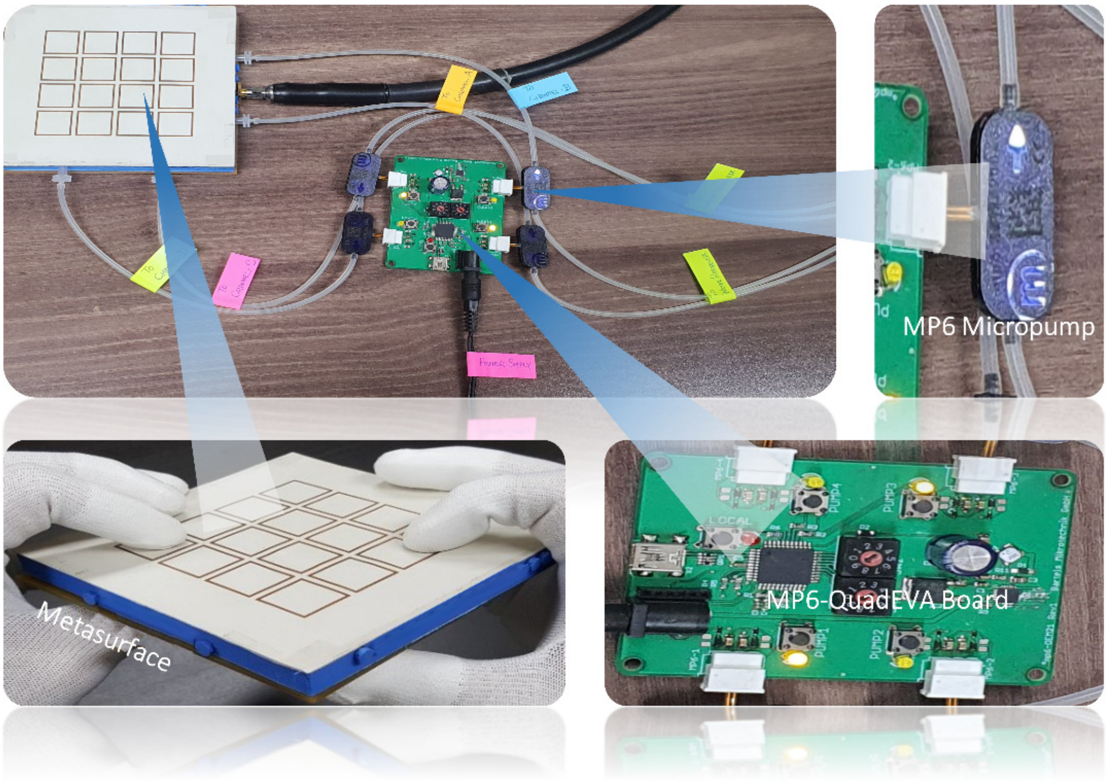

Supplementary Figure 3. Fabricated HMS prototype for experimental proof-of-concept.

Supplementary Note 4: Fabricated prototype of proposed hydrodynamic metasurface with MP6-microcontroller and micropumps

Supplementary Figure 4(a) and (b) show the simulated and measured S-parameters for all nine modes. The simulated and measured 10-dB IMBW ranged from 2.41−2.60 GHz and 2.41−2.65 GHz, respectively, for Mode-1 to Mode-9.

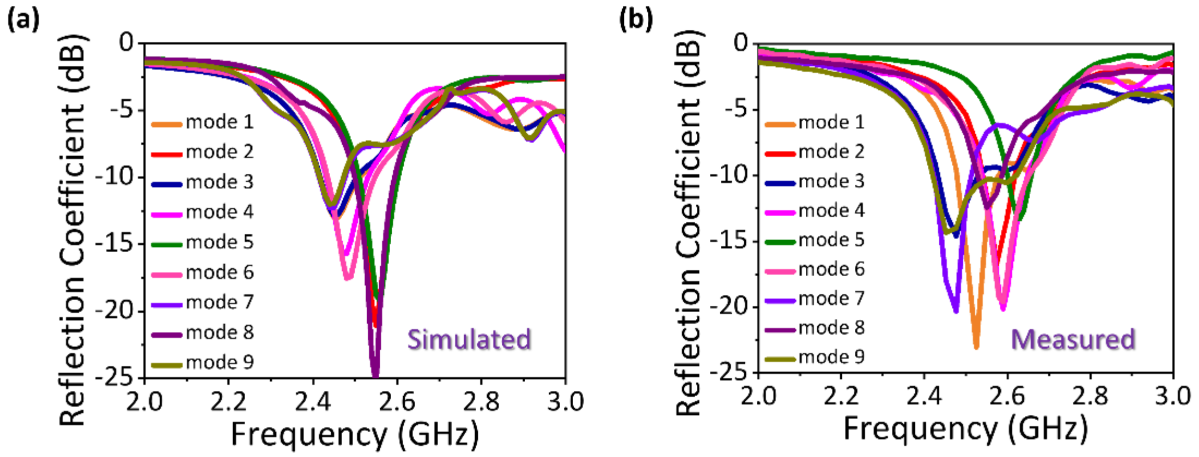

Supplementary Figure 4. (a) Simulated and (b) measured reflection coefficients for the proposed programmable hydrodynamic modes.

Supplementary Note 5: Repeatability Test

To observe the repeatability effect, we performed several experiments on the same repeating mode 6, as shown in supplementary Figure 5(a). It is observed from supplementary Figure 5(b) that the 3-dB beamwidth (HPBW) is not significantly changed while the 6-dB beamwidth is increased from 59° to 64° by repeating the measurements. In addition, supplementary Figure 5(c) shows the resonant frequency is slightly varied from 2.59 GHz to 2.55 GHz and 10-dB impedance bandwidth is varied from 2.53-2.65 GHz to 2.52-2.58 GHz by repeating the measurements. Nevertheless, 10 dB impedance matching is kept at 2.55 GHz. This variation is expected due to minor fluid leakage during 3D rotation of the prototype.

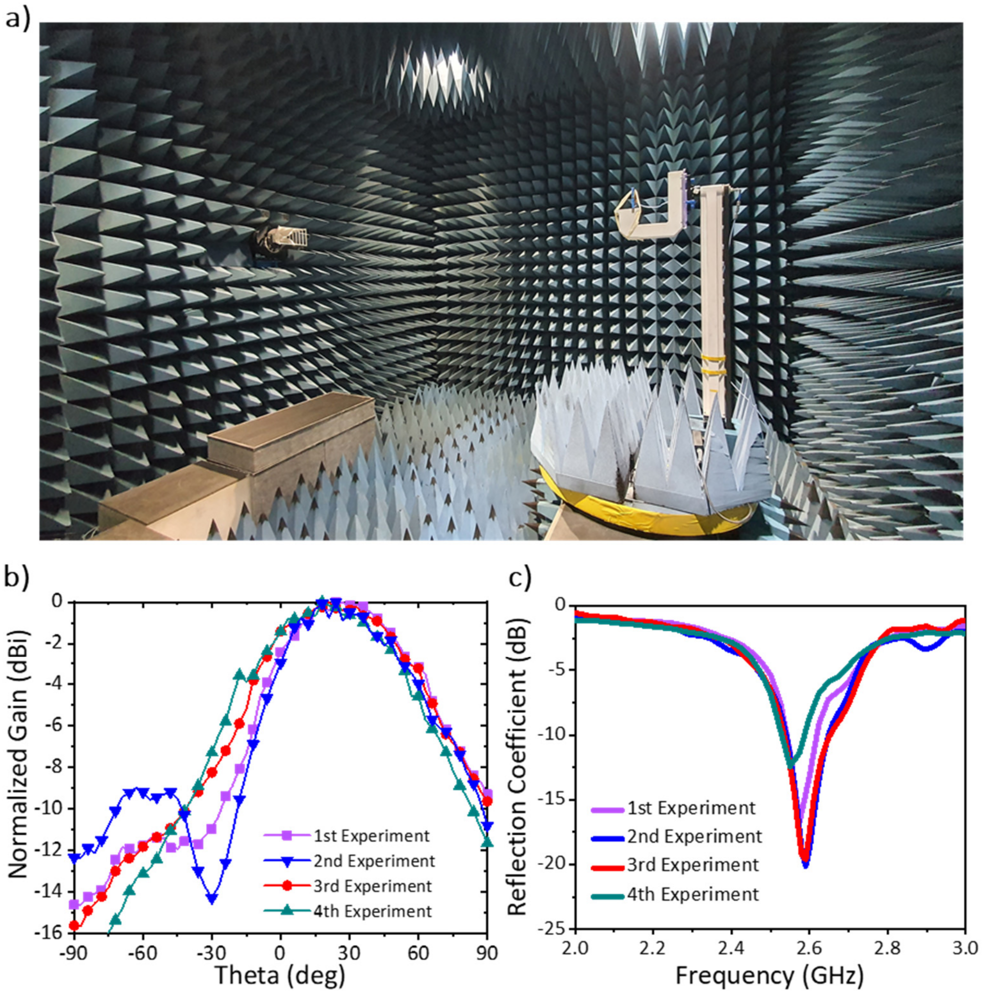

Supplementary Figure 5. (a) Photograph of measurement setup for repeatability test, (b) 2D normalized gain plots, and (c) reflection coefficient plots of mode 6 for repeatability test.
